# Retroperitoneum revisited: a review of radiological literature and updated concept of retroperitoneal fascial anatomy with imaging features and correlating anatomy

**DOI:** 10.1007/s00276-024-03432-8

**Published:** 2024-07-04

**Authors:** B. Boekestijn, M. N.J.M. Wasser, J. S.D. Mieog, M. C. DeRuiter

**Affiliations:** 1 Department of Radiology, Leiden University Medical Center, Albinusdreef 2, Leiden, 2333 ZA The Netherlands; 2 Department of Surgery, Leiden University Medical Center, Leiden, The Netherlands; 3 Department of Anatomy and Embryology, Leiden University Medical Center, Leiden, The Netherlands

**Keywords:** Anatomy, Fascia, Retroperitoneum, Imaging, Radiology

## Abstract

**Purpose:**

Spread of disease in the retroperitoneum is dictated by the complex anatomy of retroperitoneal fasciae and is still incompletely understood. Conflicting reports have led to insufficient and incorrect anatomical concepts in radiological literature.

**Methods:**

This review will discuss previous concepts prevalent in radiological literature and their shortcomings will be highlighted. New insights from recent anatomical and embryological research, together with imaging examples, will be used to clarify patterns of disease spread in the retroperitoneum that remain unexplained by these concepts.

**Results:**

The fusion fascia and the renal fascia in particular give rise to planes and spaces that act as vectors for spread of disease in the retroperitoneum. Some of these planes and structures, such as the caudal extension of the renal fascia, have previously not been described in radiological literature.

**Conclusion:**

New insights, including the various fasciae, potential spaces and planes, are incorporated into an updated combined retroperitoneal fascial concept.

## Introduction

The retroperitoneal fasciae dictate patterns of disease spread and provides barriers against invasion of malignancies such as pancreatic and renal cancer. The retroperitoneal fascial anatomy, however, is complex. Even though major fascial structures such as the posterior lamina of the renal fascia (of Zuckerkandl) and the anterior lamina of the renal fascia (of Gerota) have been described long ago, the fascial anatomy of the retroperitoneum is still incompletely understood. The tricompartmental concept (TC) is well-known in radiology and divides the retroperitoneum into three spaces [1]. However, the TC contains major flaws which are partially addressed by the more recent concept of interfascial spread (CIS). This recent concept provides a view more in line with described anatomy, in which the retroperitoneal fascial structures give rise to various planes and potential spaces [2]. The planes of the CIS, however, still conflict with anatomical and embryological studies on various aspects. Furthermore, certain fasciae and planes are not addressed in the CIS. In this article we review current knowledge of retroperitoneal fascial anatomy with an emphasis on prevalent concepts in radiological literature, address issues in current concepts and provide an updated concept of retroperitoneal anatomy by incorporating recent anatomical research, clinical imaging examples and cadaveric dissections.

### Fascial development

Before delving into the anatomy of the retroperitoneal fasciae, it is important to consider their embryological development. Three mechanisms are thought to be responsible for fascial development. Some fascial structures correspond to the sheath of connective tissue covering muscles of the abdominal wall. Examples include the fascia of the psoas major muscle (psoas fascia), of the iliacus muscle (iliac fascia) and of the transversus abdominis muscle (transversalis fascia). Some fasciae arise from the fusion of peritoneal surfaces after secondary retroperitoneal fixation. For example, the fusion fascia of Toldt corresponds to the plane between the peritoneal surface of the primary retroperitoneum and the peritoneal surface of the mesocolon after retroperitoneal fixation of the colon. The third mechanism is the migration fascia hypothesis. This hypothesis suggests that relative migration and growth of organs during embryological development induces stress, producing a linear orientation of young connective tissue fibers and therefore a fascia [3]. The prime example of a migration fascia is the renal fascia. The renal fascia only becomes apparent during embryological development after the definitive kidneys have developed and assumed their final anatomical position [4]. Thus, relative cephalic migration of the kidneys in the embryo does not seem to be solely responsible for the formation of the renal fascia and growth of various tissues also seems to play an important role.

### Current radiological concepts and their shortcomings

#### The tricompartmental concept

**Fig. 1:**
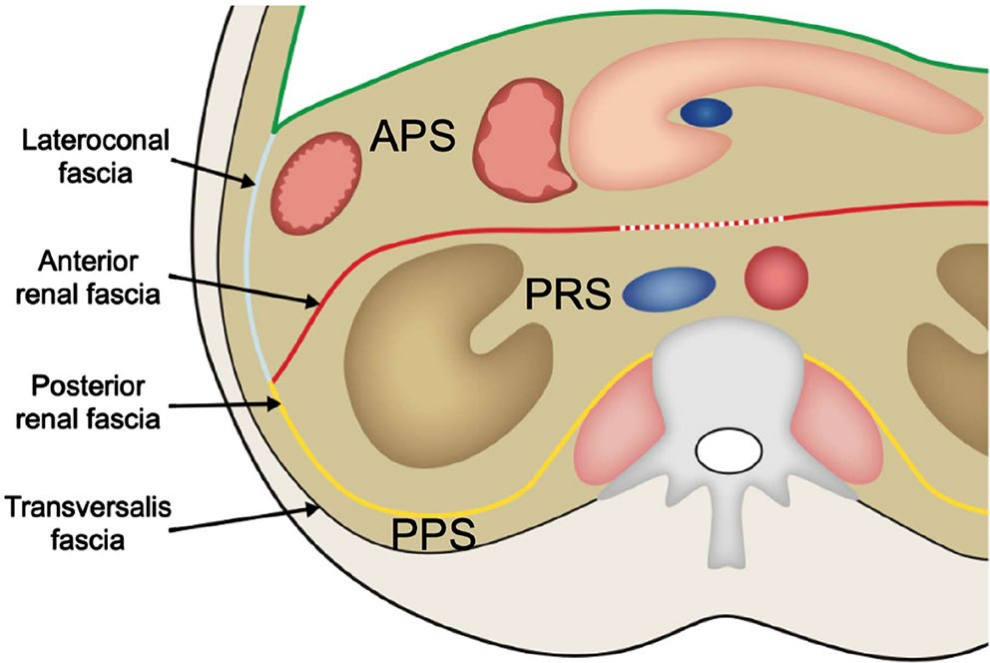
Schematic illustration of the tricompartmental concept. APS: anterior pararenal space, PRS: perirenal space, PPS: posterior pararenal space. The green line represents the peritoneum. The dotted line at the medial aspect of the anterior lamina of the renal fascia represents the supposed fusion of the fascia with connective tissue in the root of the mesentery and the dorsal aspect of the pancreas and duodenum

The TC was introduced in radiological literature by Meyers et al. in 1972 [1]. Over time this concept has become widely accepted and is recited in most radiology textbooks. This classification revolves around the renal fascia. It divides the retroperitoneum into three spaces: the perirenal, anterior pararenal and posterior pararenal space (Fig. 1). The perirenal space is the compartment in the confinement of the renal fascia, between the anterior lamina of the renal fascia (ARF) and the posterior lamina of the renal fascia (PRF). It contains the kidneys, suprarenal glands and ureters. At the medial aspect, the ARF was initially thought to blend with connective tissue surrounding the vessels in the root of the mesentery and connective tissue dorsal to the pancreas and duodenum [1]. The PRF was said to fuse with the fascia of the psoas major and the quadratus lumborum muscle [1]. The anterior pararenal space is defined as the space anterior to the ARF. It contains the secondary retroperitoneal organs: the duodenum, pancreas, ascending colon and descending colon. Lastly, the posterior pararenal space is located posterior to the PRF and is bounded by the transversalis fascia posterolaterally. The posterior pararenal space contains only adipose tissue. Sometimes a fourth compartment, the great vessel space, is also described anterior to the lumbar vertebrae containing the abdominal aorta and inferior vena cava [2, 5]. The anatomical boundaries of this “great vessel space” are not clearly defined.

**Fig. 2:**
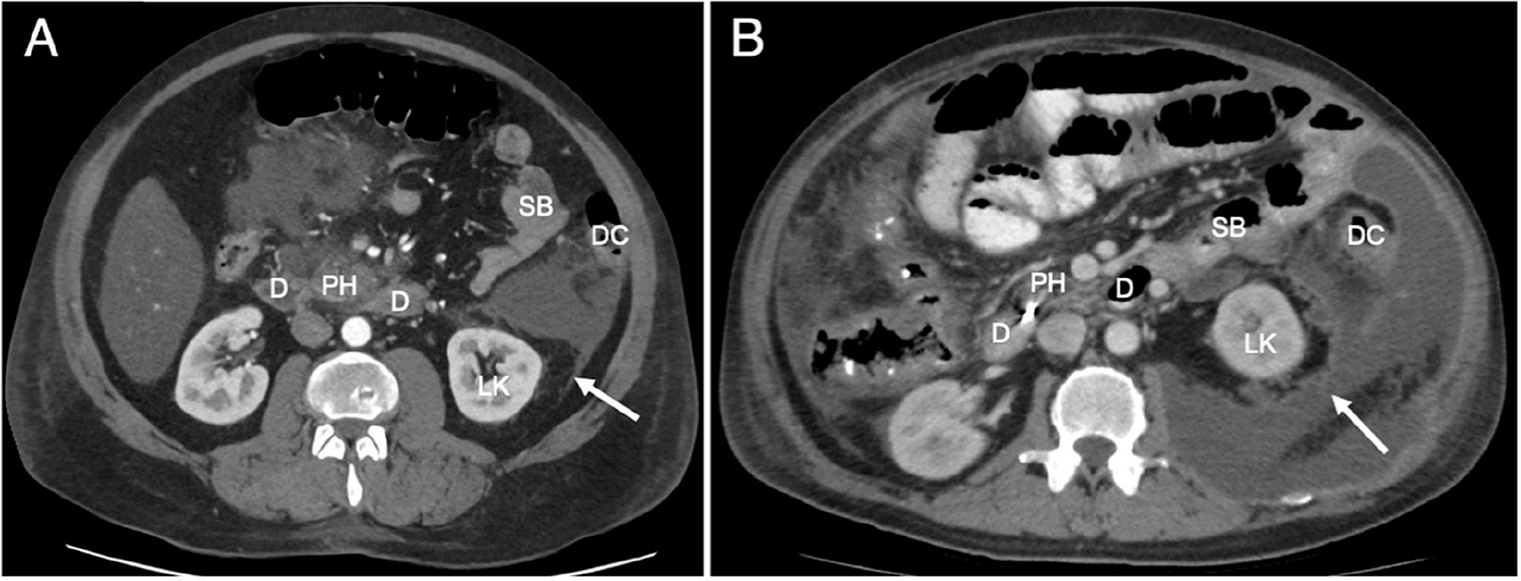
Imaging examples in CT of fluid tracking posteriorly along the renal fascia in two patients with pancreatitis. **A**) Fluid starting to spread posterior along the posterior lamina of the renal fascia (arrow). **B**) Massive amount of fluid spreading posteriorly around the renal fascia (arrow) extending medially all the way to the psoas major muscle D: duodenum, PH: pancreatic head, SB: small bowel, DC: descending colon, LK: left kidney

The TC is based on macroscopic patterns of fluid spread in the extraperitoneal space and anatomical descriptions that now are outdated. In the original concept there was thought to be no free communication between the anterior and posterior pararenal spaces due the intervening lateroconal fascia. First described in 1941 by Congdon, the lateroconal fascia was said to arise from the fusion of the lateral aspect of the ARF and PRF, continuing towards and fusing with the peritoneum of the paracolic gutter on either side [6]. The ARF, PRF and lateroconal fascia were considered to be one continuous fascial sheath, forming a boundary to spread of disease in the retroperitoneum. However, current radiological imaging provides clear examples of pathology such as retroperitoneal fluid and hematoma spreading from the anterior pararenal to the posterior pararenal space without obstruction of the lateroconal fascia (Fig. 2a, b).

**Fig. 3:**
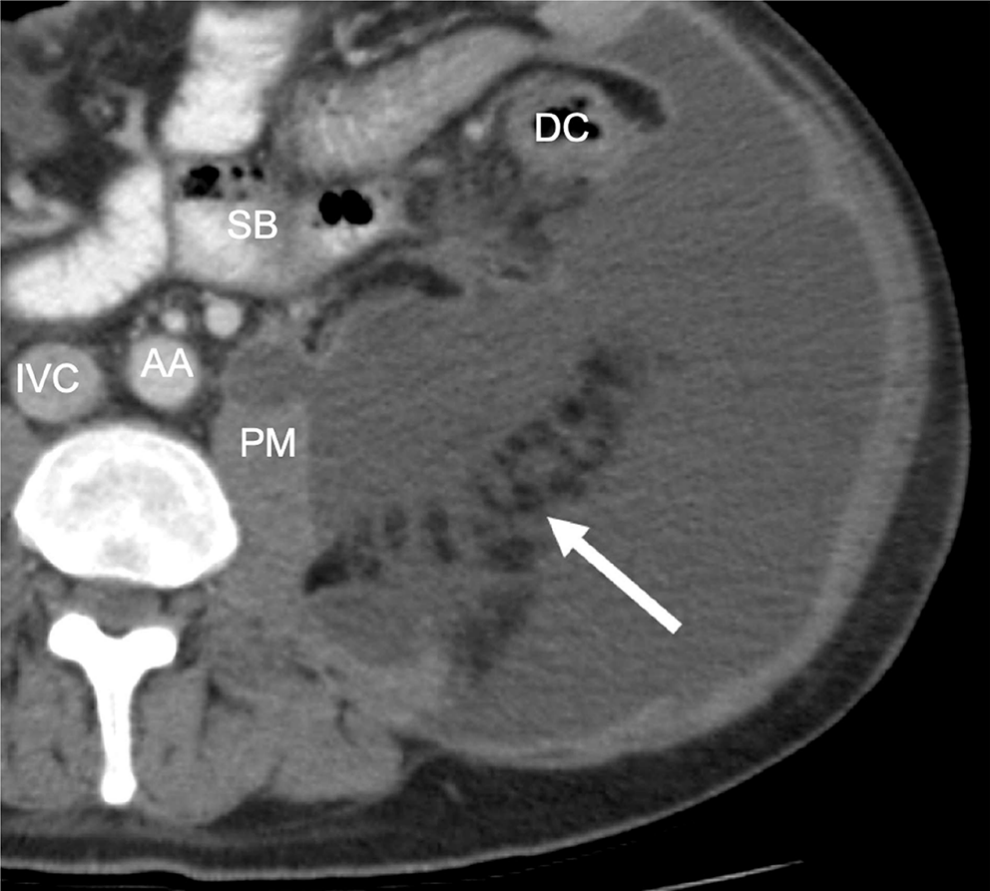
CT image of the left retroperitoneum at the level of the L4 vertebra below the kidneys. A large amount of fluid in the retroperitoneum tracks around and also dorsal to the flank pad (arrow pointing to the flank pad itself). AA: abdominal aorta, DC: descending colon, IVC: inferior vena cava, PM: psoas major muscle, SB: small bowel

Recent embryological research has demonstrated that the lateroconal fascia does not arise from fusion of the ARF and PRF, but is in fact a separate structure [7, 8]. During embryological development a body of adipose tissue arises in the lateral retroperitoneum known as the “flank pad”. Situated in the posterolateral retroperitoneum, the rapid growth of the flank pad causes the lateroconal fascia to form as a migration fascia along its anteromedial border [8]. The separate origin of the lateroconal and renal fasciae will therefore not guarantee a strict separation of the anterior and posterior pararenal spaces. This clarifies the observed patterns of fluid spread from the anterior to the posterior pararenal space. Fluid can track along the lateroconal fascia, or frankly track around and dorsal to the flank pad as is sometimes visible on imaging (Fig. 3).

Another flaw in the original TC is the anatomy of the posterior lamina of the renal fascia. As is visible in Fig. 1, the medial aspect of the fascia was thought to merge with the fascia of the psoas major and quadratus lumborum muscle. As already pointed out, the renal fascia actually is a single continuous fascial sheath encompassing both kidneys, suprarenal glands and ureters. Both the ARF and PRF cross the midline anterior to the abdominal aorta and inferior vena cava. Consequently, at the level of the renal hilum, the posterior lamina does not merge with the fascia of the muscles of the posterior abdominal wall. The posterior lamina of the renal fascia fuses with the vascular sheath of the great vessels and the renal vessels whereby the renal vessels are enveloped by the posterior lamina [8].

Finally, the TC insinuates that the secondary retroperitoneal organs (the duodenum, pancreas, ascending and descending colon) are all situated in the same compartment: the anterior pararenal space. Imaging examples, anatomy and embryology contradict this, and demonstrate the existence of additional spaces within the anterior pararenal space, which will be discussed later in this article. Some authors, however, note a separate pancreaticoduodenal and pericolic space [5].

The TC is an elegant but simplistic description of retroperitoneal anatomy not consistent with actual anatomy and patterns of disease spread.

#### The concept of interfascial spread

**Fig. 4:**
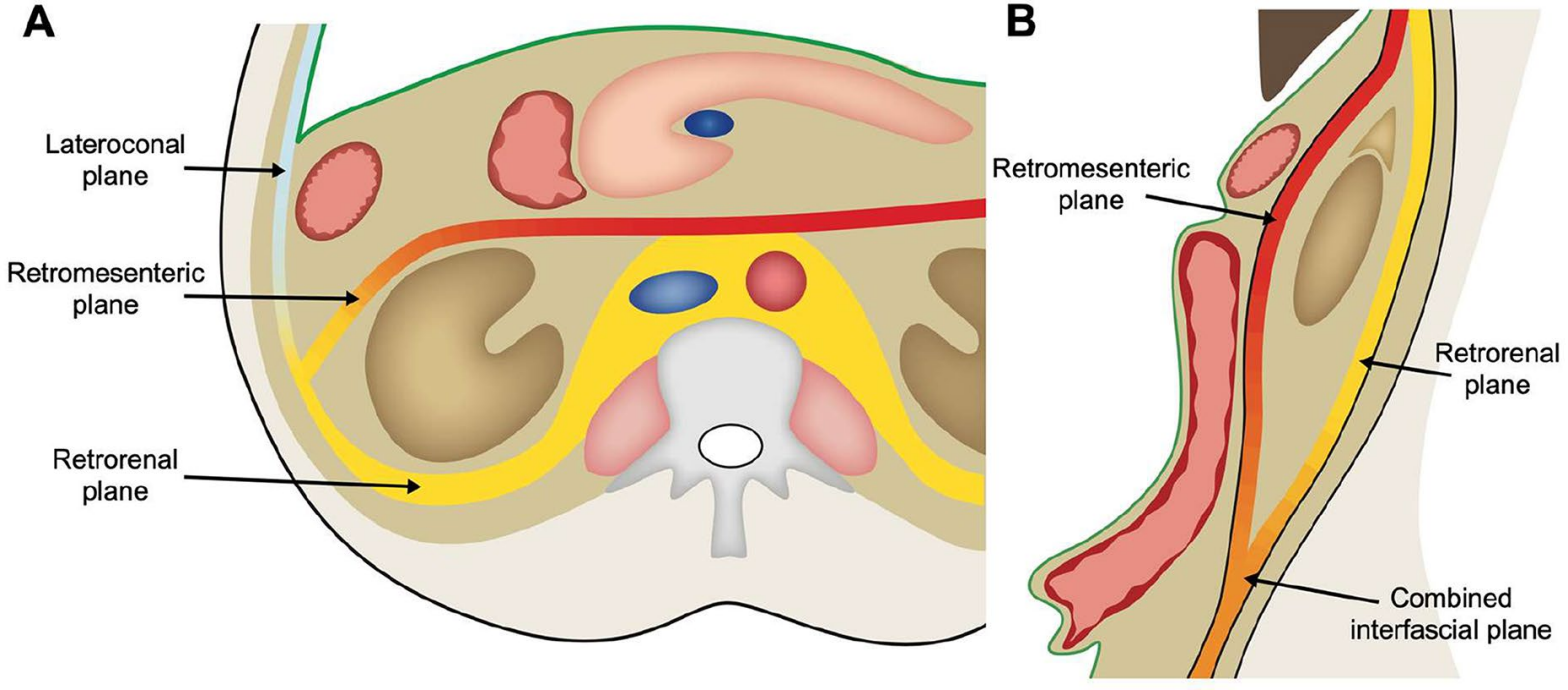
**A**) Schematic illustration of the concept of interfascial spread at the level of the pancreas in the transverse plane, showing the retromesenteric, retrorenal and lateroconal planes. These planes are continuous with each other lateral to the kidney, which is illustrated by the color gradient. The green line represents the peritoneum. **B**) Schematic illustration in the sagittal plane. The retromesenteric and retrorenal planes fuse inferior to the perirenal space to form the combined interfascial plane, illustrated by the color gradient. The peritoneum is represented in green

The TC defines different spaces in which pathology can spread. In more recent years, the CIS was introduced in radiological literature. Instead of defining spaces, this concept defines planes in between layers of retroperitoneal fasciae in which pathology can spread. The CIS was first described by Molmenti et al. [9]. Four planes are described (Fig. 4*)*: (1) the retromesenteric plane, a plane between the anterior pararenal and perirenal space; (2) the retrorenal plane, a plane posterior to the perirenal space; (3) the lateroconal plane, a plane lateral and anterior to the perirenal space in or along the lateroconal fascia; and (4) the combined interfascial plane, a plane inferior to the perirenal space where the retromesenteric and retrorenal planes fuse and create a common plane.

The CIS is based on patterns of retroperitoneal disease spread encountered on imaging. It solves some of the issues of the tricompartmental concept. For instance, it describes a plane through which disease can spread posteriorly around the renal fascia and also into the pelvis, solving one of the major shortcomings of the tricompartmental concept. However, because this concept is mostly based on imaging, it lacks proper anatomical correlation. Multiple authors attribute the interfascial planes to the fusion of the secondary retroperitoneal organs and their mesenteries with the posterior abdominal wall during embryological development, creating a multilaminar retroperitoneal fascia with different interfascial planes [9–12]. As Ishikawa et al. point out, this CIS does not correspond with actual anatomy and embryology [13]. Formation of many of the fasciae in the retroperitoneum, including the renal fascia, is not a result of fusion of the secondary retroperitoneal organs with the primary retroperitoneum, but develops as migration fasciae or are related to the abdominal wall muscles [8]. Ishikawa et al. consider the interfascial planes to be remnants of primitive loose mesenchymal connective tissue: loose connective tissue between the peritoneum and ARF constitutes the retromesenteric plane, loose connective tissue between the peritoneum and lateroconal fascia constitutes the lateroconal plane, and loose connective between the PRF and lateroconal fascia or fasciae of the posterior abdominal wall muscles constitutes the retrorenal plane. In our opinion this concept of spread in loose connective tissue provides an interpretation of the interfascial planes that is much closer to the actual anatomy. Retroperitoneal fluid travels along the path of least resistance. Organs, neurovascular structures and adipose tissue (indirectly) adhere to fasciae through loose connective tissue. Loose connective tissue is easily separated to form a potential space. This means there is a potential plane in loose connective tissue through which fluid and other pathology can spread, instead of planes only existing in between two apposed fasciae as the original literature of the CIS suggests [11]. Rather than thinking of the retroperitoneum as multiple fasciae and several anatomical planes, the retroperitoneum should be envisioned as a continuous extraperitoneal space with multiple fasciae and structures along which pathology can spread. This conceptualization is commonplace in anatomical literature, but lacking in radiological literature and clinical radiology practice.

**Fig. 5:**
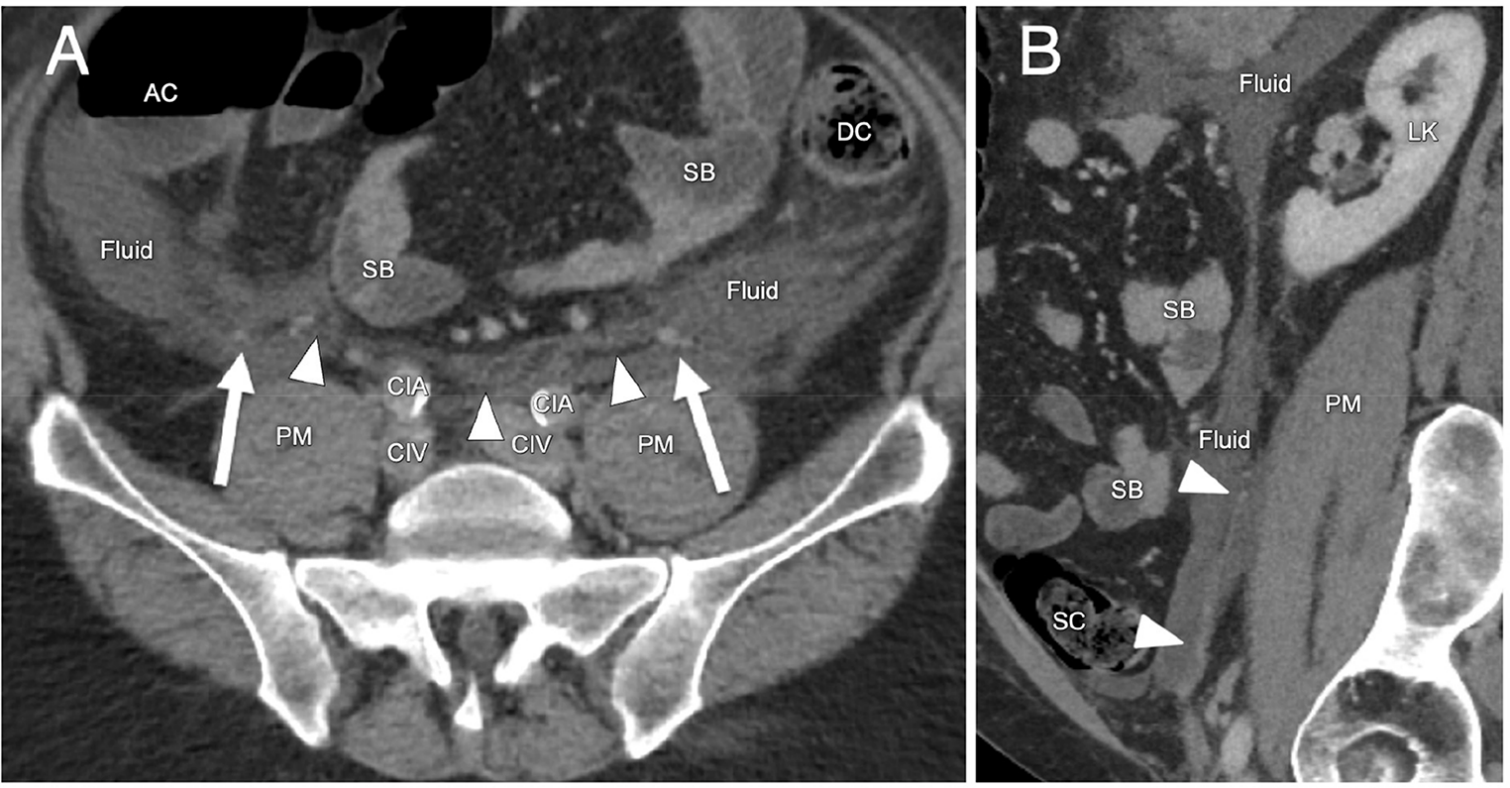
Retroperitoneal fluid in a patient with pancreatitis demonstrated on CT imaging. **A**) At the level of the pelvis fluid tracks around the caudal extension of the renal fascia which is visible as a bilateral sheath of adipose tissue. The gonadal vessels (arrows), mark the approximate lateral border of the caudal extension of the renal fascia. **B**) Sagittal reconstructions demonstrate the caudal extension of the renal fascia (arrow heads), containing the ureter and testicular vessels coursing to the inguinal canal. AC: ascending colon, CIA: common iliac artery, CIV: common iliac vein, DC: descending colon, LK: left kidney, PM: psoas major muscle, SB: small bowel, SC: sigmoid colon

The CIS presents several shortcomings. It describes the combined interfascial plane below the level of the kidneys, where the retromesenteric and the retrorenal planes are said to merge. However, this is not in line with the anatomy of the renal fascia. Caudal to the kidneys, the renal fascia remains one continuous structure crossing the midline and extending into the pelvis. At this level, the renal fascia encompasses the ureters, gonadal vessels and perinephric veins over their entire retroperitoneal course [8, 14]. Thus, caudal to the level of the kidneys these structures are still contained in between the ARF and PRF. The gonadal vessels are the most laterally located structures within the caudal extension of the renal fascia and therefore can demonstrate the approximate lateral boundary of the renal fascia. The caudal extension of the renal fascia is sometimes visible on imaging and can be verified by the position of the ureters and gonadal or perinephric vessels (Fig. 5*).* Fluid anterior to the caudal extension of the ARF can only reach posterior to the PRF by tracking laterally around the renal fascia. This means that a combined interfascial plane only exists lateral to the renal fascia, which to our knowledge has not been reported in the literature previously. Therefore, the term “combined interfascial plane” arguably is a misnomer. The caudal extension of the renal fascia reaches deep into the pelvis where its lateral border is at the deep inguinal ring and it medially envelops the urinary bladder [15].

It is interesting to note that recent research suggests the majority of the perirenal fat belongs to the connective tissue embryologically related to the gonad and suprarenal gland. Only the renal hilar fat and a small amount periureteral fat is related to the urinary system and is encapsulated by a sheath of connective tissue known as the “ureteral sheath” [14]. This is in accordance with the anatomy of the vessels in the perirenal space. The gonadal veins anastomose with the perinephric veins, which in turn anastomose with the suprarenal veins [14].

**Fig. 6:**
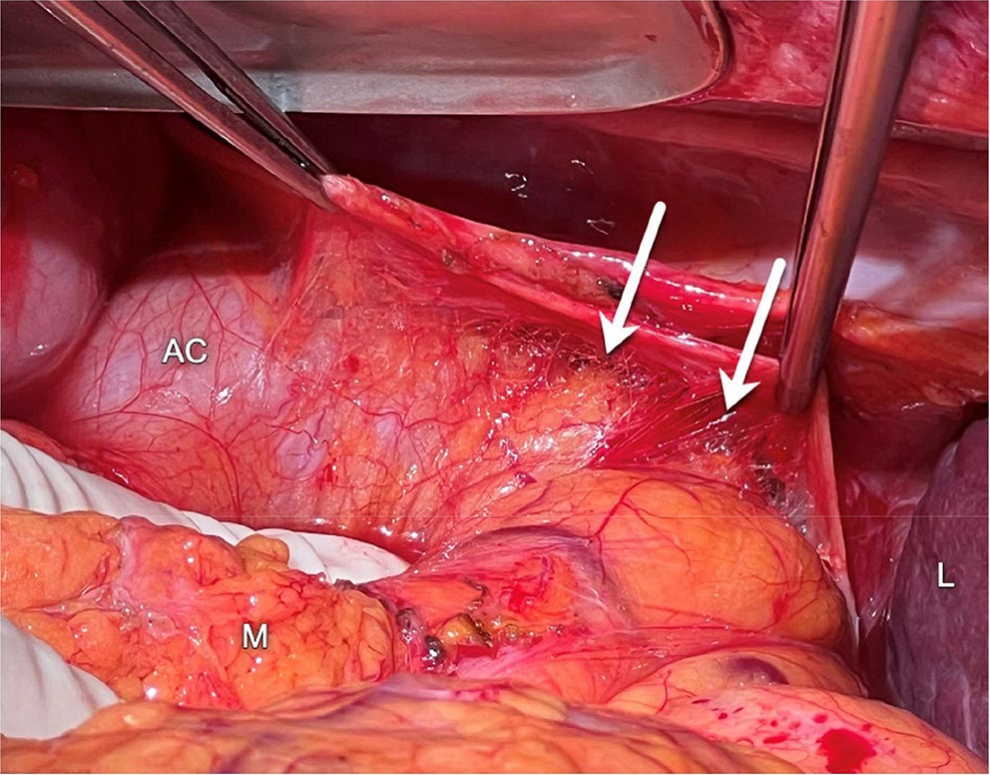
Loose connective tissue around the ascending colon visible during surgery as thin irregular strands (arrows). AC: ascending colon, L: liver, M: mesentery

**Fig. 7:**
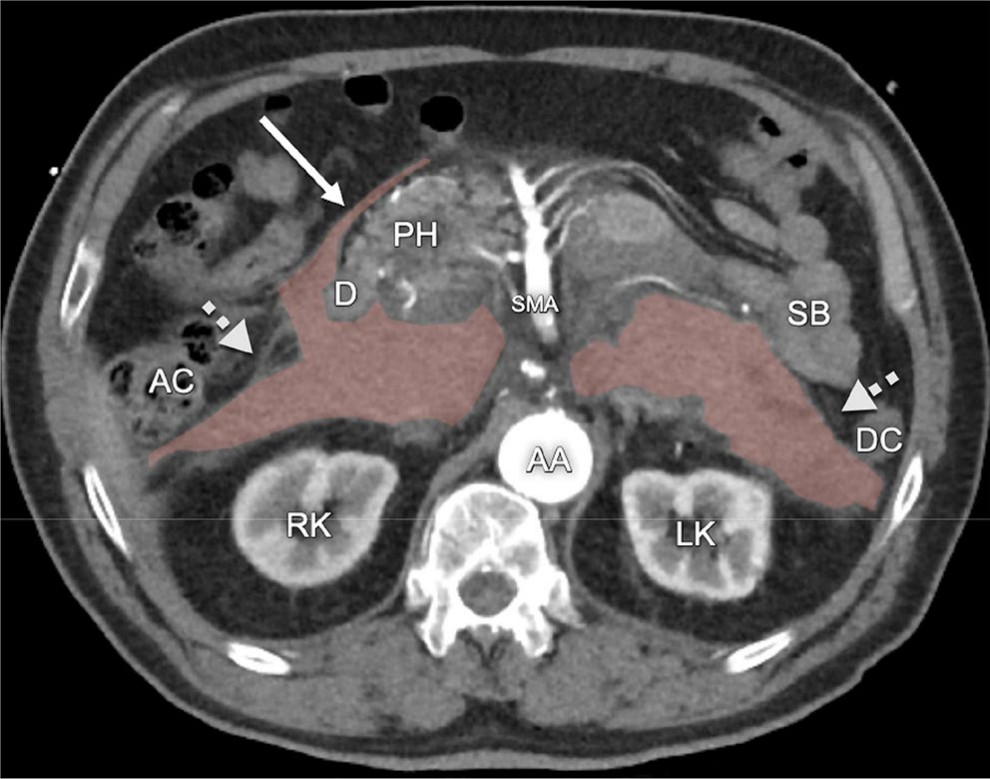
Patient with a retroperitoneal hematoma due to a ruptured abdominal aortic aneurysm. The hematoma (highlighted in red) spreads in a plane dorsal to the mesocolon (dotted arrow bilaterally marks the position of adipose tissue in the mesocolon) and also extends in a plane anterior to the pancreatic head within the fusion plane of Fredet (arrow). AA: abdominal aorta, AC: ascending colon, D: duodenum (descending part), DC: descending colon, LK: left kidney, RK: right kidney, SB: small bowel, SMA: superior mesenteric artery, PH: pancreatic head

Another shortcoming of the CIS is the lack of compartmentalization of the secondary retroperitoneal organs. The pancreas and duodenum and are not located in the same compartment as the ascending and descending colon. The secondary retroperitoneal organs develop as intraperitoneal organs during fetal development, possessing a free dorsal mesentery. During development these organs and their mesenteries fuse with the dorsal wall of the peritoneal cavity, producing a secondary retroperitoneal fixation. Fusion of the duodenum and pancreas occurs first, well before fixation of the ascending and descending colon. Along these planes of fusion, several famous anatomists have described fusion fasciae: the *fascia of Toldt* [16] dorsal to the ascending and descending colon and their mesenteries; the *fusion fascia of Treitz* [17] dorsal to the pancreas (retropancreatic fascia); and the *fusion fascia of Fredet* [18] between the ventral aspect of the pancreatic head, duodenum and the ascending mesocolon. Multiple authors consider the retromesenteric plane to correspond to the potential space in the fusion fascia between the mesenteries of the secondary retroperitoneal organs and the primary retroperitoneum [9–12]. Though, such a plane would not be able to communicate with the retrorenal plane, as Ishikawa et al. also point out [13]. They further state that after fusion the mesothelial cells of the opposed peritoneal surfaces disappear, producing a single inseparable layer. However, recent anatomical studies in adults have shown remnants of both the visceral and parietal layer of the opposed peritoneal surfaces of the ascending and descending colon and their mesenteries, with loose connective tissue in between corresponding to the fascia of Toldt [19, 20]. These planes are used by surgeons as bloodless dissection planes in mobilization of the colon, mesocolon, duodenum and pancreas [18]. The loose connective tissue present in these planes allows for movement that occurs under physiological circumstances. During surgery, loose connective tissue in the planes and around structures can easily be separated by blunt dissection (Fig. 6*)*. Imaging examples appear to demonstrate the spread of disease in these planes as well. For instance, spread of disease can be seen in a plane dorsal to the mesocolon (the fascia of Toldt), extending dorsal to the pancreatic head (the fusion fascia of Treitz) and further extending ventral to the pancreatic head (the fusion fascia of Fredet) (Fig. 7*)*. Extension of disease in a plane ventral to the pancreatic head cannot be explained by the retromesenteric plane of the CIS, but is in keeping with the anatomy of the fusion planes. Furthermore, disease spreading in a plane dorsal to the mesocolon but not extending into the retrorenal plane, as is visible in Fig. 6, also is consistent with the anatomy of the fusion planes.

### Updated concept of retroperitoneal fascial anatomy

**Fig. 8:**
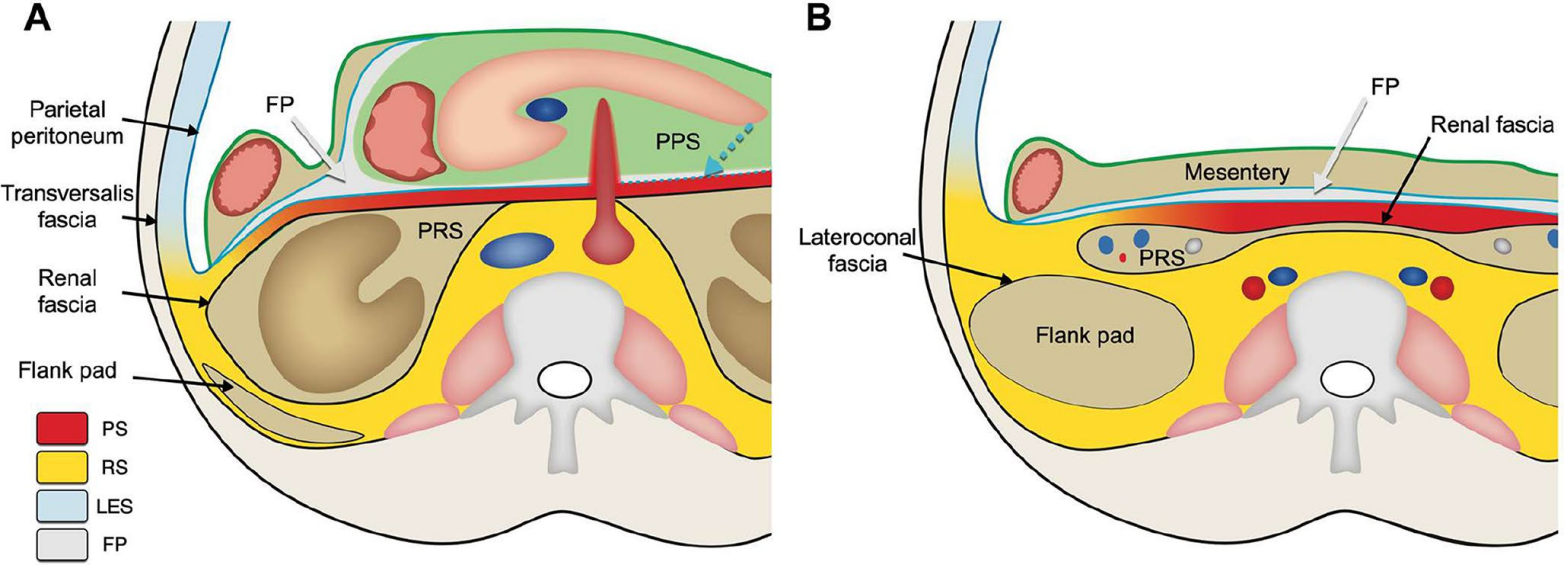
(**A**) Combined retroperitoneal fascial concept at the level of the pancreas. PS: prerenal space; RS: retrorenal space; LES: lateral extraperitoneal space; FP: fusion plane; PRS: perirenal space; PPS: peripancreatic space. The prerenal and retrorenal spaces, as well as the lateral extraperitoneal space, are continuous with each other indicated by the color gradient. The fusion plane (light gray) is present in between remnants of the peritoneum (light blue). The retropancreatic peritoneal remnant is illustrated as a dotted line (also marked with a dotted light blue arrow) to the left side of the superior mesenteric artery because of its close relationship and potential fusion with the anterior lamina of the renal fascia. (**B**) Combined retroperitoneal fascial concept at a level inferior to the kidneys. PS: prerenal space; RS: retrorenal space; LES: lateral extraperitoneal space; FP: fusion plane; PRS: perirenal space (caudal extension containing the ureters and gonadal vessels). The ‘flank pad’ is the major body of retroperitoneal adipose tissue at this level. The lateroconal fascia along the anteromedial border of the flank pad is represented by the thickened black line

In this section we provide an updated radiological concept of retroperitoneal fascial anatomy, which addresses the shortcomings of the TC and the CIS. Recent anatomical and embryological research will be discussed, which combined with observed patterns of disease spread on imaging, forms the basis of this updated view. Figure 8 illustrates our combined retroperitoneal fascial concept, including retroperitoneal fasciae, spaces and potential planes.

In this combined retroperitoneal fascial concept, we integrate current knowledge of the fusion planes, the peripancreatic space and the caudal extension of the renal fascia into previous concepts. Spread of disease can grossly be divided into (1) spread in the primary retroperitoneum, (2) spread in the fusion planes and (3) spread in the secondary retroperitoneal compartments.

#### 1. Spread of disease in the primary retroperitoneum

The primary retroperitoneum is divided by the renal fascia in the perirenal space (within the boundaries of the renal fascia) and the main retroperitoneal space. In this main space, disease can spread in loose connective tissue along adipose tissue and along the retroperitoneal fasciae. The main retroperitoneal space extends from the diaphragm to the pelvic diaphragm and extends along the abdominal wall into the preperitoneal space. Three compartments can be defined in the main retroperitoneal space:

- The *prerenal space* (PS), located anterior to the anterior lamina of the renal fascia and bounded by remnants of the peritoneum anteriorly, after fixation of the secondary retroperitoneal organs and their mesenteries. It merely contains loose connective tissue and a small amount of adipose tissue. The PS crosses the midline and extends caudally into the pelvis, because of the caudal extension of the renal fascia. Due to the close proximity of the of the anterior renal fascia to the remnants of the peritoneum, the space is very thin. In the midline the prerenal retroperitoneal space communicates with loose connective tissue in the root of the mesentery. As the renal fascia courses all the way to the deep inguinal ring and urinary bladder, this space extends caudally until the urinary bladder.
- The *retrorenal space* (RS), located posterior to the posterior lamina of the renal fascia and bounded by the fascia of the quadratus lumborum (anterior lamina of the thoracolumbar fascia) and psoas muscles (iliopsoas fascia) posteriorly, as well as the anterior longitudinal ligament covering the lumbar vertebrae. The RS also crosses the midline. The space contains adipose tissue, loose connective tissue, nerves related to the lumbosacral plexus, lymphatics and blood vessels such as the lumbar vessels. The abdominal aorta and inferior vena cava are included in this space because both the anterior and posterior laminae of the renal fascia cross the midline anterior to the great vessels. The retrorenal space extends inferiorly into the pelvis and is continuous with the presacral space.
- The pre- and retrorenal spaces merge with each other lateral to the boundary of the renal fascia into a *lateral extraperitoneal space* (LES). The posterolateral border of this space is formed by the transversalis fascia. The anteromedial border is the parietal peritoneum or peritoneal remnants of the ascending and descending colon, depending on whether the space has passed the lateral border of the ascending or descending colon. The lateral LES is continuous with the preperitoneal space along the abdominal wall. Like the RS, the LES contains adipose tissue, loose connective tissue, nerves related to the lumbosacral plexus, lymphatics and small blood vessels. The LES also extends into the pelvis where it reaches until the deep inguinal ring.

The retroperitoneal adipose tissue of the flank pad, which is covered by the lateroconal fascia on its anteromedial aspect, is located at the border of the RS and LES. Spread of disease usually occurs along the anterior aspect of the flank pad, but in some cases can spread posterior to the flank pad (see Figs. 2 and 3*for examples*).

#### 2. Spread of disease in the fusion planes

The *fusion planes* (FP) are the potential planes in the loose connective tissue of the fusion fascia. The FP is situated in between peritoneal remnants of the parietal peritoneum of the posterior abdominal wall and remnants of the visceral peritoneum of the secondary retroperitoneal organs and their mesenteries prior to fixation. These planes therefore are situated dorsal to the ascending colon, descending colon, pancreas, duodenum and their fixated mesenteries. Along the lateral border of the descending part of the duodenum, the plane extends ventrally around the pancreatic head (fusion fascia of Fredet).

#### 3. Spread of disease in the secondary retroperitoneal compartments

**Fig. 9:**
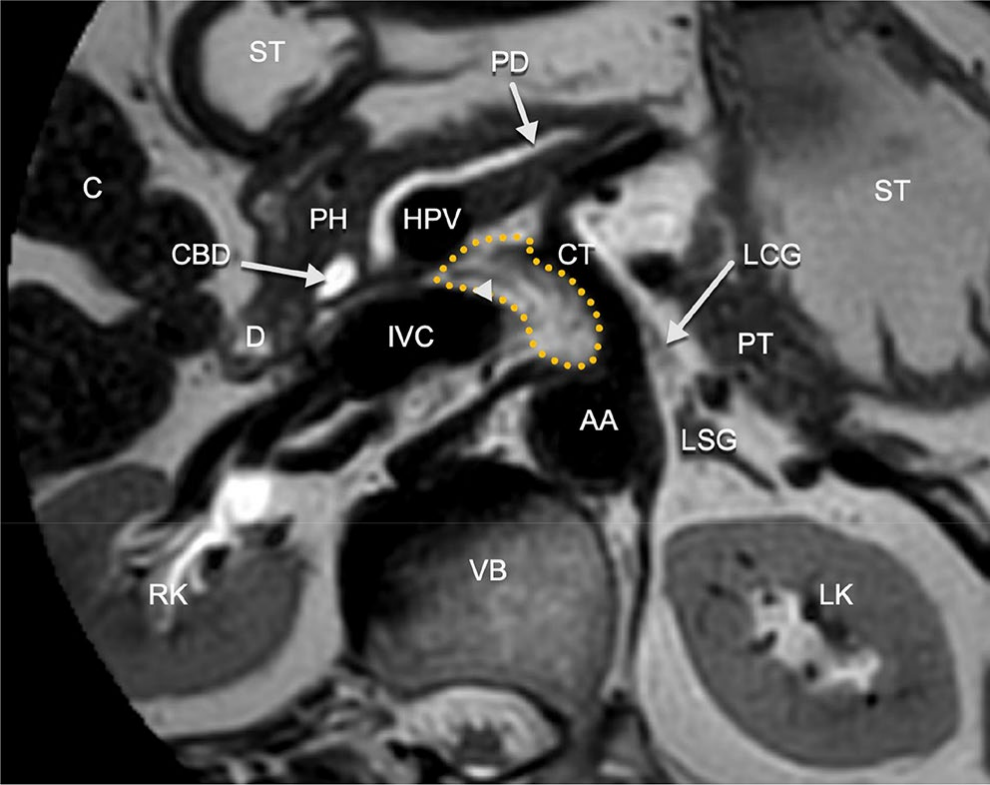
T2-weighted magnetic resonance image of the pancreatic head. The neural plexus contained by the retropancreatic fascia is visible (dotted yellow area in which several linear structures (arrow head) are visible corresponding to nerve fibers of the plexus). The left celiac ganglion (LCG) is visible to the left side of the celiac trunk (CT). AA: abdominal aorta, C: colon, CBD: common bile duct, D: duodenum (descending part), HPV: hepatic portal vein, IVC: inferior vena cava, LK: left kidney, RK: right kidney, LSG: left suprarenal gland, PD: pancreatic duct, PH: pancreatic head, PT: pancreatic tail, ST: stomach, VB: vertebral body

The development of the fusion fascia dorsal to the pancreatic head was investigated extensively by Cho et al. [4]. Retroperitoneal fixation of the pancreatic head occurs at about 10 weeks gestation, at which it is connected to the retroperitoneum by loose connective tissue without an observable remnant of the peritoneum. At 20 weeks gestation a distinctive fascia develops dorsal to the pancreatic head and duodenum, which is separated from the developing renal fascia by loose connective tissue. By 25 weeks gestation the retropancreatic fascia develops further and two laminae could be recognized, one adhering to the duodenum and one in proximity to the renal fascia coursing laterally. Cho et al. thereby state that the fascia of Treitz is not merely a fusion fascia, but represents the transformation of an indistinct remnant of the peritoneum into a distinct fascia. According to these findings, the retropancreatic fascia could therefore be seen as a combination of a fusion and migration fascia. Spread of disease may occur in the plane of this fascia and course laterally into the fusion fascia of Toldt dorsal to the mesocolon and course anterior in the fusion fascia of Fredet along the pancreatic head, as seen in Fig. 7. The retropancreatic fascia may also act as a barrier to infiltration by pancreatic cancer [21]. The rarity of invasion of the inferior vena cava by tumors of the pancreatic head coincides with this [22], even though the pancreatic head is usually situated against the anterior aspect of the vena cava. All the neurovascular structures are contained between the pancreatic parenchyma and the fascia. On imaging, the fusion fascia is not clearly visible. The vessels and nervous plexus contained between the pancreatic parenchyma and the retropancreatic fascia, however, are visible on high resolution magnetic resonance images (Fig. 9*).*

**Fig. 10:**
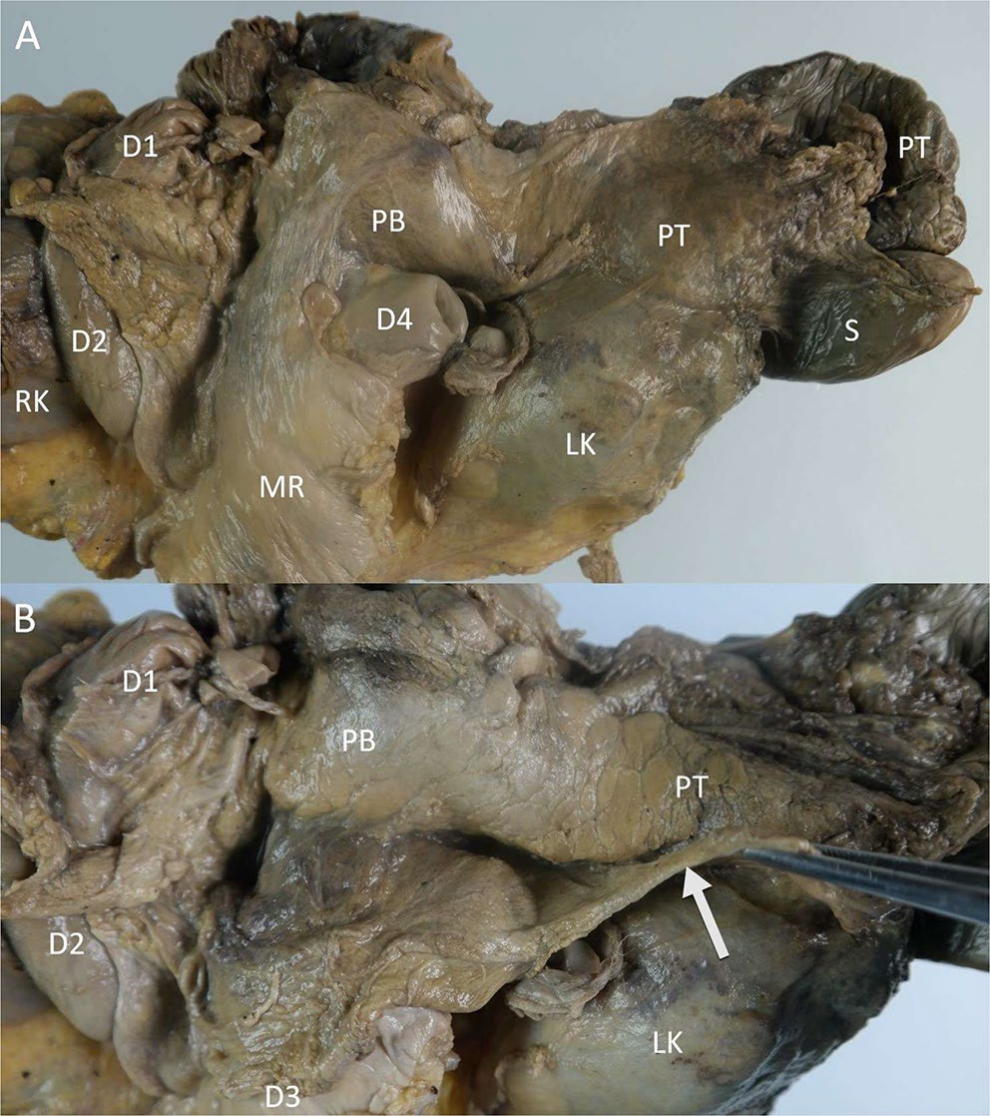
Photographs of the pancreas during dissection on a specimen from a Fix for Life © embalmed human cadaver. **A**) The ventral side of the pancreas is still covered by its peritoneal covering.**B**) The peritoneal covering is detached from the pancreatic body and tail and pulled caudally (arrow), revealing the pancreas itself and the peripancreatic space. D1: duodenal bulb, D2: descending part of the duodenum, D3: horizontal part of the duodenum, D4: ascending part of the duodenum, LK: left kidney, MR: mesenteric root, PB: pancreatic body, PT: pancreatic tail, S: spleen

The peripancreatic space is another area of interest, not addressed in previous radiological concepts of fascial anatomy. The peritoneal covering and retropancreatic fusion fascia envelop the pancreas and provide the boundaries of the peripancreatic space (Fig. 10*)*. During pancreatitis, inflammatory fluid and necrotic collections often form in the peripancreatic space. The retropancreatic fascia is incomplete at the level of the superior mesenteric artery, allowing for communication with the primary retroperitoneal space [23]. The inferior part of the pancreatic head is covered by the root of the mesentery and the fixation of the transverse mesocolon. At the level of the pancreatic body and tail, the attachment of the transverse mesocolon runs at the level of, or inferior to the pancreas. Due to the close relationship to the mesenteric root and transverse mesocolon, peripancreatic disease often spreads into these mesenteries.

The retropancreatic fascia dorsal to the pancreatic body and tail has not been investigated as extensively as the fascia at the level of the pancreatic head. At the pancreatic body and tail, the retropancreatic fascia is in close proximity to the ARF. Yang et al. reported only a retropancreatic fascia at the level of the pancreatic body and tail, without an evident ARF [23]. During embryological development the ARF is situated close to the parietal peritoneum, sometimes fusing with the latter [8]. The retropancreatic fascia at the level of the pancreatic body and tail was recently investigated in near term fetuses by Kim et al. [24]. They found a variable retropancreatic fascia. Some fetuses demonstrated a multilaminar retropancreatic fascia of which the posterior lamina corresponded to the renal fascia. Other fetuses, however, demonstrated a single thick fascial layer possibly caused by the fusion of the renal fascia with remnants of the peritoneum. They also found a variable configuration of the fusion fascia of the ascending and descending mesocolon, sometimes also demonstrating a multilaminar aspect. The patterns of fluid spread in pancreatitis reflect these findings. Peripancreatic fluid collections often extend into the prerenal space at the level of the pancreatic tail, possibly due to the close relation and variable fusion of the retropancreatic fascia with the ARF. These differing reports on the retropancreatic fascia show that there is no single accepted view of its composition.

**Fig. 11:**
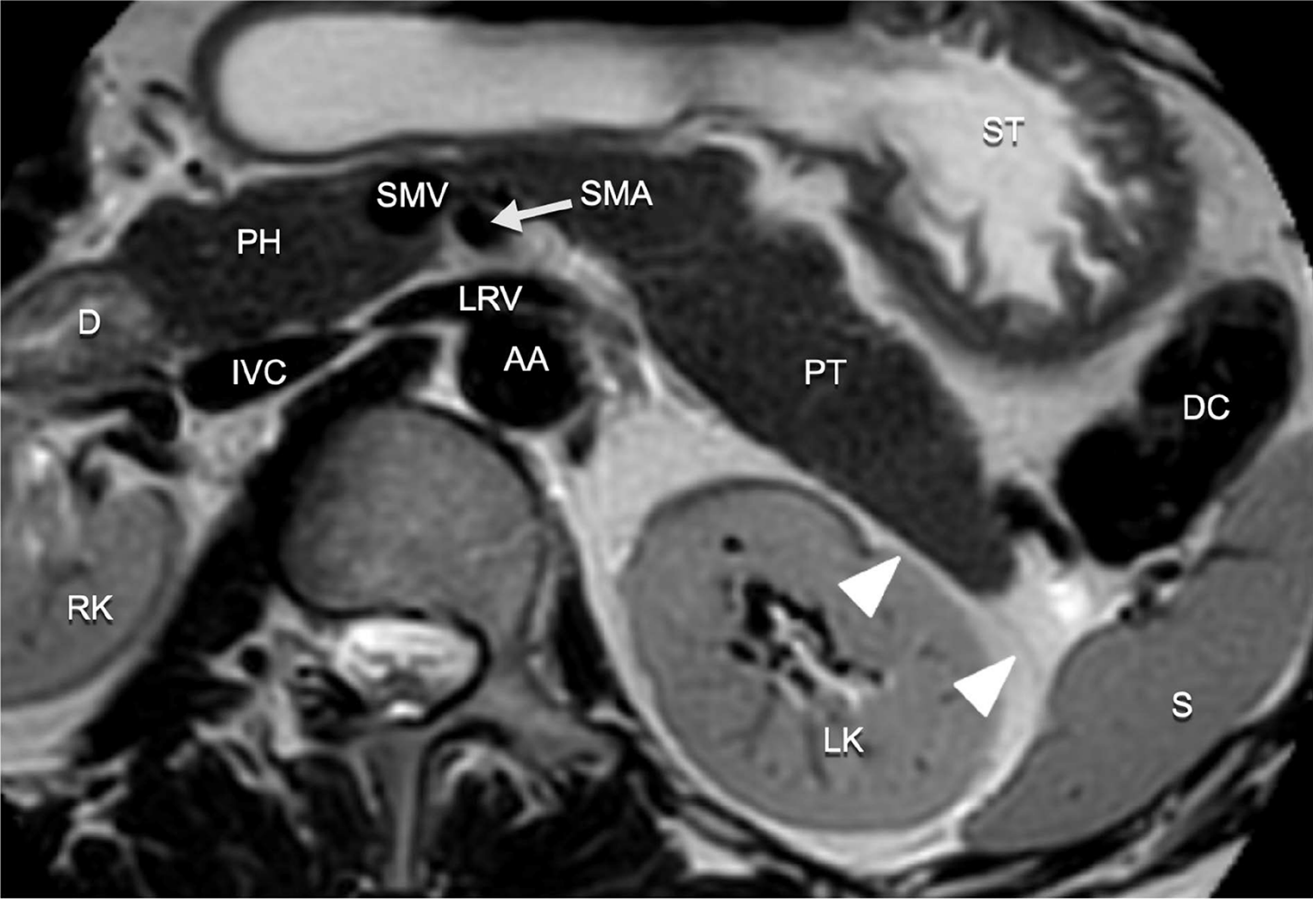
T2-weighted MR image of the pancreatic tail. The anterior renal fascia (arrow heads) is in close proximity to the pancreatic tail. AA: abdominal aorta, D: duodenum (descending part), DC: descending colon, IVC: inferior vena cava, LK: left kidney, LRV: left renal vein, PH: pancreatic head, PT: pancreatic tail, RK: right kidney, S: spleen, SMA: superior mesenteric artery, SMV: superior mesenteric vein, ST: stomach

The fusion fascia dorsal to the pancreatic body and tail is not clearly visible on imaging, the ARF is in close proximity to the pancreatic tail (Fig. 11*).* At the level of the pancreatic tail, the fusion fascia may also provide a barrier to tumor infiltration of pancreatic cancer [21]. However, at the level of the superior mesenteric artery and vein the fascia is absent providing a route for direct tumor invasion [23]. Perineural invasion is a hallmark of pancreatic cancer [25]. The location of the neural plexus and the absence of the fascia around the vessels contribute to pattern of tumor spread towards and along the major peripancreatic vessels.

**Fig. 12:**
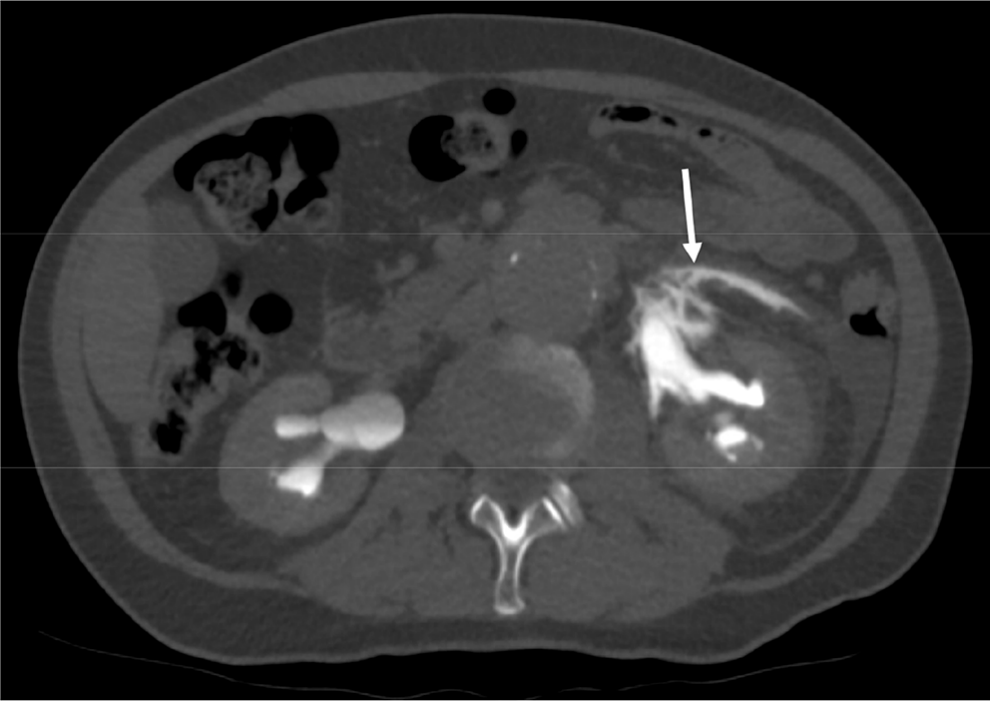
A patient with blow-out of the renal pelvis due to retroperitoneal fibrosis. An excretory phase at CT shows leakage of contrast from the renal pelvis into the perirenal space and also extending through the ARF into the prerenal space (arrow). The focal discontinuation of the fascia is most likely pathological due to the presence of the retroperitoneal fibrosis

It is important to realize that the anatomy of the retroperitoneal fasciae is variable. Some fasciae can be multilaminar or fused into a single layer. Therefore, the retroperitoneal planes may not consistent between individuals and different patterns of disease spread can occur. Furthermore, the fasciae can be disrupted by pathology (Fig. 12). Also, in some area’s fasciae can be incomplete. For example, the superior aspect of the renal fascia may allow for communication between the perirenal space and the bare area of the liver on the right, and the left subphrenic recess [26]. Thus, sometimes it may be impossible to attribute spread of disease on imaging to a particular plane or space. However, this possible uncertainty does not mean that an understanding of the complex fascial anatomy is not worthwhile. For radiologists, recognizing a certain pattern of disease spread can help immensely with making the correct diagnosis that otherwise may not have been made as quickly. For example, spread of disease in the fusion plane is often pancreatic or duodenal in origin. Presence of hematoma in the fusion plane in the context pancreatitis should prompt the search for vascular injuries such as pseudoaneurysms, often requiring additional imaging with CT angiography.

## Conclusion

The anatomy of the retroperitoneal fasciae, potential planes and spaces is complex and often misunderstood due to the abundance of conflicting reports in the literature. Previous concepts of retroperitoneal fascial anatomy presented in radiological literature were reviewed and expanded upon with recent anatomical insights, resulting in an updated concept of fascial anatomy and spread of disease. An improved understanding of fascial anatomy may help radiologist in correctly diagnosing the origin of retroperitoneal pathology.

## Data Availability

No datasets were generated or analysed during the current study.
